# Transport Pathways—Proton Motive Force Interrelationship in Durum Wheat Mitochondria

**DOI:** 10.3390/ijms15058186

**Published:** 2014-05-09

**Authors:** Daniela Trono, Maura N. Laus, Mario Soccio, Donato Pastore

**Affiliations:** 1 Consiglio per la Ricerca e la sperimentazione in Agricoltura, Centro di Ricerca per la Cerealicoltura, S.S. 673 Km 25, 71122 Foggia, Italy; E-Mail: daniela.trono@entecra.it; 2 Dipartimento di Scienze Agrarie, degli Alimenti e dell’Ambiente, Università di Foggia, Via Napoli 25, 71122 Foggia, Italy; E-Mails: maura.laus@unifg.it (M.N.L.); mario.soccio@unifg.it (M.S.)

**Keywords:** plant mitochondria, potassium channel, anion channel, ADP/ATP carrier, uncoupling protein, mitochondrial carriers, membrane potential, reactive oxygen species

## Abstract

In durum wheat mitochondria (DWM) the ATP-inhibited plant mitochondrial potassium channel (PmitoK_ATP_) and the plant uncoupling protein (PUCP) are able to strongly reduce the proton motive force (pmf) to control mitochondrial production of reactive oxygen species; under these conditions, mitochondrial carriers lack the driving force for transport and should be inactive. However, unexpectedly, DWM uncoupling by PmitoK_ATP_ neither impairs the exchange of ADP for ATP nor blocks the inward transport of Pi and succinate. This uptake may occur via the plant inner membrane anion channel (PIMAC), which is physiologically inhibited by membrane potential, but unlocks its activity in de-energized mitochondria. Probably, cooperation between PIMAC and carriers may accomplish metabolite movement across the inner membrane under both energized and de-energized conditions. PIMAC may also cooperate with PmitoK_ATP_ to transport ammonium salts in DWM. Interestingly, this finding may trouble classical interpretation of *in vitro* mitochondrial swelling; instead of free passage of ammonia through the inner membrane and proton symport with Pi, that trigger metabolite movements via carriers, transport of ammonium via PmitoK_ATP_ and that of the counteranion via PIMAC may occur. Here, we review properties, modulation and function of the above reported DWM channels and carriers to shed new light on the control that they exert on pmf and *vice-versa*.

## 1. Introduction

Mitochondria play a key role in all aerobic eukaryotic cells, as the site of respiration and ATP synthesis via oxidative phosphorylation. In addition to cellular energy supply, mitochondria provides, through the tricarboxylic acid cycle, reducing equivalents for other compartments and carbon skeletons for biosynthesis of nucleotides, amino acids, fatty acids and vitamin cofactors. In plants, mitochondria are also involved in plant-specific metabolic pathways, including nitrogen assimilation, photorespiration, and photosynthesis in C4 and CAM plants, as well as utilization of carbon and nitrogen storage compounds during seed germination [1–4]. So, for the maintenance of basic energy function and the complex metabolic network involving mitochondria and other cell compartments, a rapid and controlled movement of solutes in and out of mitochondrion is required. The flux of hydrophilic solutes across the inner mitochondrial membrane, that represents a diffusion barrier generally impermeable to charged and polar molecules, is facilitated and regulated by a large number of specific hydrophobic transport proteins, including carriers and ion-conducting channels.

Currently, with respect to plant mitochondrial transporters in plants, several metabolite transporters of inner mitochondrial membrane have been identified at the molecular level and recognized as members of the mitochondrial carrier family (MCF) in light of their structural features similar to their counterparts from animals and yeasts [4]. However, some of these plant MCF genes have not yet been unambiguously associated with a function and with a defined physiological role in plant metabolism [2,3]. In sequenced plant genomes the number of genes encoding putative MCF proteins varies from 37 to 125, thus being larger than that of *Saccharomyces cerevisiae* and comparable with that of *Homo sapiens*. In particular, in *Arabidopsis thaliana* 58 MCF genes have been reported. On the basis of substrate specificity, four functional subfamilies of the proteins of the MCF have been recently proposed [4,5].

The first subfamily comprises nucleotide and nucleotide derivate transporters [4]. In *Arabidopsis thaliana* three ADP/ATP carriers (AAC1–3) have been identified, which are responsible for the electrogenic export of ATP^4−^ generated in the mitochondrial matrix via oxidative phosphorylation in counter-exchange with cytosolic ADP^3−^ [6–8]. Recently, in the same plant species, the ADNT1 carrier has been identified as responsible for the counter-exchange of mitochondrial ATP with cytosolic AMP, and only to a lesser extent ADP [9]; moreover, three putative ATP/Pi carriers (APC1-3) are supposed to catalyze ATP provision by facilitating the electroneutral exchange between MgATP^2−^ and Pi^2−^ [5]. Two NAD^+^ transporters (NDT1-2) mediate the fast import of the adenylated cofactor into mitochondria in counter-exchange with various nucleotides (preferentially ADP and AMP), as well as a slow unidirectional NAD^+^ transport [10]. Moreover, evidence has been reported of the existence of two mitochondrially located MCF proteins related to the mitochondrial thiamine pyrophosphate carriers (TPC) of animals and *Saccharomyces cerevisiae* [11], as well as of two mitochondrial CoA transporters [12].

The second subfamily comprises the proteins of the MCF that mediate the passage through the inner mitochondrial membrane of di- and tricarboxylates or ketoacids [4]. In *Arabidopsis thaliana* these proteins include three dicarboxylate carriers (DIC1–3) [13], a dicarboxylate/tricarboxylate carrier (DTC1) [14] and a specific succinate/fumarate carrier (SFC1) [15,16].

A third functional MCF subfamily includes transporters for amino acids, carnitine and *S*-adenosylmethionine [4]. In *Arabidopsis thaliana* two basic amino acid carriers (BAC1–2) have been identified: BAC1, involved in arginine/ornithine exchange during mobilization of storage proteins in germinating seeds [17,18], and BAC2, responsible for the transport of arginine, ornithine and citrulline and, therefore, involved in proline, polyamine and alkaloid synthesis or in the shuttling of ammonia between mitochondria and plastids [17–20]. As far as the carnitine transport, the BOU1 carrier identified in *Arabidopsis thaliana* has been hypothesized to import acetyl-carnitine (generated in peroxisomes from acetyl-CoA) in exchange for carnitine to directly feed the Krebs cycle with acetyl units [21]. *Arabidopsis thaliana* also possess two transporters (SAMC1 and SAMC2), which mediate the specific import of *S*-adenosylmethionine exclusively synthesized in the plant cytosol and act as a donor of methyl-groups, in counter-exchange with *S*-adenosylhomocysteine [22,23].

Carriers responsible for the transport of other substrates, like uncoupling proteins (UCP) and Pi carriers (PiC), constitute the fourth MCF subfamily [4]. In *Arabidopsis thaliana* six MCF sequences encode plant UCPs (PUCP1–6), which catalyze a nucleotide-sensitive and free fatty acid (FFA)-mediated re-entry of protons into matrix, resulting in an energy dissipation [24–27]. Two proven and one putative Pi carriers (PiC1–3) facilitates an electroneutral ΔpH-driven Pi/OH^−^ antiport or Pi/H^+^ symport, so allowing Pi supply required for ATP synthesis and for metabolite uptake by means of other MCF proteins [28].

Functional, structural and evolutionary aspects of plant MCF carriers are dealt in detail in some recent reviews [3–5,7].

Along a large number of carriers, the inner membrane of plant mitochondria also contains ion-conducting channels that endow it with controlled permeability to small ions [29]. Plant inner membrane ion channels have been functionally characterized using both classical bioenergetic measurements and electrophysiological techniques, but very little information is available about their molecular identity. The electrogenic transport of K^+^ ions into the mitochondrial matrix has been described in different plant species and is mediated by selective potassium channels exhibiting different biochemical and electrophysiological properties: ATP-regulated and ATP-insensitive K^+^ channels; large conductance Ca^2+^-activated and Ca^2+^-insensitive K^+^ channels [30,31]. It should be considered that the electrophoretic K^+^ influx through potassium channels is coupled to K^+^ extrusion through an electroneutral K^+^/H^+^ exchanger [32,33]. As for other cations, Ca^2+^-transporting systems have been proposed in plant mitochondria [34,35] although they are elusive to date. Recently, a major mitochondrial iron transporter, essential for plant growth and development, has been identified and characterized in rice [36,37]. With respect to anion conductance pathways, the occurrence of a plant inner membrane anion channel (PIMAC), catalyzing the electrophoretic uniport of different anions, has been functionally demonstrated in mitochondria from a few species [38–40]; a chloride channel activity has been described in potato [41], as well as chloride channel protein performing PIMAC activity has been identified in maize [42].

As to the driving-force dependence of mitochondrial transporters, it should be remembered that substrate oxidation by the mitochondrial respiratory chain is accompanied by protons ejection in the intermembrane space, thus generating the proton motive force (pmf). Pmf represents the driving force not only for ATP synthesis but also for many protein-mediated transport activities across the inner mitochondrial membrane. Pmf consists of two components: the electrical membrane potential (ΔΨ), which is the main component in plant mitochondria ([1], and references therein), and the proton gradient (ΔpH), which is lower than in mammalian organelles.

In recent years, our research group has studied different transport pathways in durum wheat mitochondria (DWM), thus showing the occurrence of an electrogenic K^+^ uptake via PmitoK_ATP_ channel acting together with a K^+^/H^+^ exchanger; a fatty acid-mediated H^+^ transport via Plant Uncoupling Protein (PUCP); a PIMAC-like anion-conducting pathway; an ATP/ADP exchange and a Pi import via ATP/ADP and Pi carriers, respectively; a succinate/malate exchange, probably involving the DICs carriers and/or SFC; and, finally, a malate/oxaloacetate exchange, expected to involve DICs. Moreover, a proline uptake has been described in DWM by Di Martino *et al.* [43]. In most of these papers, attention has been made on functioning of these transport systems in relation to driving force. Here, we summarize current knowledge on DWM channels and carriers in comparison with other plant mitochondria, focusing attention on the interrelationship between transport activities and pmf in a physiological perspective. In particular, two aspects are addressed: (i) pmf decrease due to the operation of dissipative transport systems (PmitoK_ATP_ channel and PUCP); (ii) functioning of ΔΨ- and ΔpH-driven transport pathways in conditions of decreased pmf. This second aspect is of particular interest since it is poorly understood.

## 2. PmitoK_ATP_, PUCP and ΔΨ

### 2.1. PmitoK_ATP_ and K^+^/H^+^ Antiporter

DWM represent the source in which a protein-mediated K^+^ uptake has been described for the first time in plant mitochondria [44]. Its existence was assessed firstly by using swelling technique and by following channel-dependent ΔΨ changes [44], and it has been successively confirmed by means of the first patch clamp study on plant mitochondria [45]. In these mitochondria, K^+^ uptake is mediated by an ATP-inhibited channel named plant mitoK_ATP_ (PmitoK_ATP_) by analogy to the possible animal counterpart, the mitoK_ATP_ [46]. Several other mitochondrial potassium channels have been described since then; a cyclosporine A-activated and ATP-inhibited mitochondrial potassium channel was described in pea [47], in soybean suspension cell cultures [48], in three coniferous species [49,50] and in *Arum* spadix and tubers [51]. A quinine-inhibited but ATP-insensitive potassium channel was instead reported in potato, maize and tomato mitochondria [52]. Other less characterized K^+^ pathways were reported by our research group in mitochondria from bread wheat, spelt, rye, barley and spinach [44], potato [53], topinambur, triticale, lentil and *Arabidopsis thaliana*. By using an electrophysiological approach based on planar lipid bilayer technique, three potassium channels were described in potato tuber mitochondria: a large conductance Ca^2+^-activated and iberiotoxin-sensitive potassium channel, namely the mitoBKCa [54], an ATP-sensitive mitoK_ATP_ and a large-conductance Ca^2+^-insensitive and iberiotoxin-sensitive channel [41].

**Figure 1.**
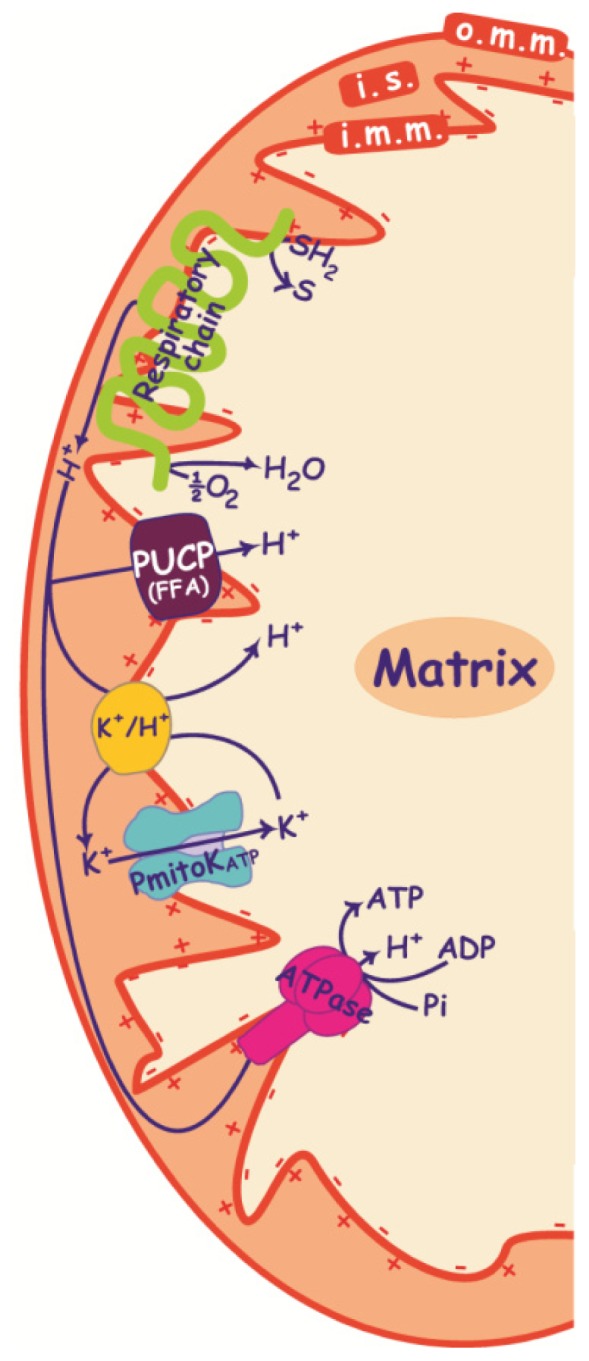
PmitoK_ATP_ and PUCP functioning in DWM. Proton ejection into the intermembrane space by the respiratory chain, which generates a proton electrochemical gradient, is indicated. This proton motive force (pmf) consists of two components: the electrical membrane potential or ΔΨ (+ and − signs in the figure), which is the main component of pmf in plant mitochondria, and the proton gradient or ΔpH, which is even negligible in DWM. The proton re-entry into the matrix via the ATPase drives the ATP synthesis. PmitoK_ATP_ catalyses the electrophoretic K^+^ uptake across the inner membrane towards the matrix; when K^+^ uptake via PmitoK_ATP_ is associated with a K^+^ efflux through the K^+^/H^+^ antiporter, a very active K^+^ cycle is generated, that allows proton re-entry into the matrix. PUCP mediates proton re-entry into the matrix in the presence of free fatty acids (FFAs). SH_2_, reduced substrates; S, oxidized substrates; i.m.m., inner mitochondrial membrane; i.s., intermembrane space; o.m.m., outer mitochondrial membrane.

DWM also possess a very active K^+^/H^+^ antiporter [44]. In DWM, PmitoK_ATP_ catalyses the electrophoretic uniport of K^+^ through the inner mitochondrial membrane; the co-operation between PmitoK_ATP_ and the K^+^/H^+^ antiporter allows the operation of a K^+^ cycle (Figure 1). In the course of substrate oxidation, protons are ejected in the intermembrane space by complexes I, III and IV of the respiratory chain, thus generating the pmf; the K^+^ cycle causes re-entry of protons in the mitochondrial matrix, that strongly reduces both ΔΨ and ΔpH ([44], and references therein). As regards the operation of the K^+^ cycle in DWM, the PmitoK_ATP_ is very different from other mitochondrial potassium channels.

Recently, an investigation carried out on isolated potato tuber mitochondria demonstrated that potato mitoK_ATP_ activity caused a ΔΨ decrease of only few mV (up to 2.5 mV) [41]. This different behavior between DWM and potato tuber mitochondria may depend on the different experimental systems used. In potato tuber mitochondria experiments, NADH was used as respiratory substrate and ΔΨ decrease was measured using TPP^+^ in an indirect manner, *i.e.*, as difference between measurements carried out in the presence of diazoxide (a mitoK_ATP_ activator) plus ATP and that carried in the presence of diazoxide alone; on the contrary, in DWM experiments, succinate was the respiratory substrate and the probe safranin O was used to directly measure KCl-induced ΔΨ decrease (see [55] for a discussion about these differences). However, it should be underlined that potato mitoK_ATP_ activity, evaluated by the same protocol employed for DWM, was found to be lower with respect to DWM-PmitoK_ATP_ [44]. This difference may also depend on the phylogenetic distance between potato (a dicotyledonous) and durum wheat (a monocotyledonous).

Similarly to potato tuber mitochondria, in heart mitochondria the increased K^+^ influx associated to potassium channel opening was small and it was able to depolarize by only 1–2 mV [56]; this is probably due to the fact that, in mammalian mitochondria, the maximal rate of the K^+^ cycle, given by the Vmax of the K^+^/H^+^ antiporter, is only about 20% of the maximal rate of proton ejection [57].

In DWM, PmitoK_ATP_ presents a conductance of 150 pS in 150 mM KCl [45]; how this low conductance may be related to ability of the channel to strongly affect ΔΨ remains to be established. As for potato tuber mitochondria, the mitoBKCa shows a conductance ranging from 502 to 615 pS [54]; for the ATP-sensitive mitoK_ATP_ and the large-conductance Ca^2+^-insensitive and iberiotoxin-sensitive channel, a conductance of 164 and 312 pS was measured, respectively [41].

Moreover, DWM-PmitoK_ATP_ shows a strong voltage dependence, with activity that remains constant between 95 and 140 mV and rapidly increases from 140 to 175 mV [44]. Although under physiological conditions, the channel is expected to mainly transport K^+^; it may work as an ATP-sensitive cation channel showing low selectivity for K^+^ toward Na^+^ in patch clamp experiments [45]. On the other hand, swelling and ΔΨ experiments show some selectivity: Cs^+^ > K^+^ = Rb^+^ > Na^+^ = Li^+^ [44]. Interestingly, in DWM the channel has been reported to actively transport NH^4 +^ (see Section 3.1), thus suggesting that it has other potentially novel physiological roles. As for potato tuber mitochondria, by using ΔΨ experiments, a high selectivity for K^+^ toward Na^+^, Li^+^, Cs^+^ and Rb^+^ was measured for all three K^+^ channels [41,54].

Like mammalian mitoK_ATP_, DWM-PmitoK_ATP_ is inhibited by ATP and ADP, and ATP inhibition is prevented or reversed by GTP and diazoxide; the ATP inhibition of DWM-PmitK_ATP_ is noncompetitive with a Ki value equal to 290 μM as evaluated by ΔΨ measurements [44]. Consistently, patch-clamp technique shows half inhibition at 500 μM ATP [45]. It should be noted that in DWM PmitoK_ATP_ affinity for ATP is significantly lower (about 10–15-fold) than the mitoK_ATP_ found in rat liver and beef heart mitochondria [46]. In DWM, ATP inhibition of PmitoK_ATP_ is independent on the presence of Mg^2+^ ions [44], while ATP inhibition of mammalian mitoK_ATP_ exhibits an absolute requirement for Mg^2+^ [58]. Interestingly, DWM-PmitoK_ATP_ is insensitive to glyburide, that is known to inhibit K^+^_ATP_ channel activity in mammalian mitochondria [59], as well as in plant mitochondria obtained from pea stems [60] and potato tubers [41]. DWM-PmitoK_ATP_ is also stimulated by the sulfydryl group reagents mersalyl and *N*-ethylmaleimide (NEM). As for physiological modulators other than ATP, DWM-PmitoK_ATP_ activity is decreased by NADH and mainly increased by superoxide anion, which induces an approximately 100% increase in the PmitoK_ATP_ activity [44]. FFAs also stimulate PmitoK_ATP_ activity by 2–4-fold, and an even higher increase (5–12-fold) occurs in the presence of their acyl-CoA ester derivatives [61], in contrast to mammalian mitoK_ATP_ that is inhibited by palmitoylCoA [58].

For the K^+^ channel activities detected in plant mitochondria, the information available on the molecular identity is still very scarce. Although a series of hypotheses have been reported [45], to the best of our knowledge, only a congress report is available about the partial purification of a potato mitoK_ATP_, that has been proposed to contain Kir and SUR type subunits [62], and the recognition, by a proteomic approach, of a β regulatory subunit of a non-identified voltage-gated K^+^ channel in rice mitochondria [63]. More information is available about the animal channel counterpart with the interesting recent identification of the renal outer medullary potassium channel (ROMK, Kir1.1) as a subunit of the mitoK_ATP_ channel in bovine heart mitochondria ([64], and references therein).

### 2.2. PUCP

The uncoupling protein (UCP) is a specialized protein that catalyses the FFA-mediated, purine nucleotide-inhibited re-entry of protons across the inner mitochondrial membrane, thus uncoupling the electron transport along the respiratory chain and the ATP synthesis by the ATP synthase (Figure 1).

In mammals, five distinct isoforms have been identified as members of the UCP family: the UCP1, found exclusively in brown adipose tissue and responsible for the adaptive thermogenesis, the ubiquitous UCP2, the skeletal muscle-specific UCP3, and the brain-specific UCP4 and brain mitochondrial carrier protein (BMCP1) (for review see [24]).

For plants, before the discovery of any mammalian UCP other than UCP1, Vercesi and coworkers demonstrated in 1995 the existence of a UCP in potato mitochondria, and named it PUMP (plant uncoupling mitochondrial protein) [65]. To avoid a possible confusion with the term “pump” used for transporters responsible for active transport, the acronym PUMP has been successively replaced with PUCP (plant uncoupling protein). Since the discovery of the first UCP homologue in plants, the existence of this mitochondrial protein has been demonstrated in a variety of organs and tissues of higher plant species including monocots and dicots, and C3, C4, and CAM plants [27,66]. As said above, upon the completion of the genome sequencing, six genes that encode PUCPs have been identified in *Arabidopsis thaliana* [25]; beside *Arabidopsis thaliana*, UCP homologue cDNAs have been identified in the dicot species mango, tomato and the thermogenic *Aracea* ([66], and references therein). As far as monocots, five PUCP expressed sequences have been identified in the sugarcane EST database [25]; PUCP cDNAs have been also isolated from rice [67], wheat [68] and maize [69]. Sequence analysis has revealed that PUCP are phylogenetically closer to UCP2 and UCP3 than to UCP1.

As far as DWM, the existence of an active PUCP has been demonstrated more than a dozen years ago [70]. In these mitochondria, calculation of the PUCP proton conductance values were carried out as proton leak rate/ΔΨ considering only the ATP-sensitive portion [71]. In fact, a part of uncoupling may be due to alternative pathways insensitive to ATP, such as non-protein membrane pores or protein/lipid interfaces (“proton leak”), or transporters other than the PUCP, *i.e.*, aspartate/glutamate antiporter, PiC and AAC, which are known to mediate the FFA cycling; so, PUCP is responsible for partial ΔΨ depolarization. However, in DWM, linoleate, one of the most abundant unsaturated fatty acids in plants, is able to cause a fast and complete dissipation of ΔΨ when added to DWM at a concentration ranging between 5 and 12 μM and the linoleate-induced ΔΨ dissipation in DWM is largely recovered by the addition of 0.5 mM ATP, a powerful PUCP inhibitor [70], thus demonstrating high activity of PUCP.

So, the above reported findings demonstrate that, with the notable exception of UCP1 in brown adipose tissue [72], the DWM-PUCP is probably the most active uncoupling protein so far described among animal and plant mitochondria. The rate of linoleate-induced ΔΨ decrease shows the typical features of a protein-mediated transport, with a sigmoidal saturation dependence as a function of linoleate concentration and a K_0.5_ of 16 μM; a substrate specificity, with a preference for unsaturated fatty acids; a dependence on ΔΨ, with a largely enhanced rate at ΔΨ values between 120 and 130 mV [70]. In addition, DWM-PUCP shows features typical of other known mammalian and plant UCPs [24], such as inhibition by ATP, GTP, GDP and Mg^2+^ [70].

Interestingly, DWM-PUCP has been the first uncoupling protein for which evidence of activation by reactive oxygen species (ROS) has been reported; indeed, our findings demonstrated an increase (about 40%) in the rate of linoleate-induced depolarization in the presence of either hydrogen peroxide or superoxide anion, this latter produced *in situ* by the xanthine/xanthine oxidase system [70]. Successively, evidence has been reported that the activation by ROS represents a general feature of UCPs from both mammalian [73–76] and plant sources [77,78], and that it occurs indirectly, through lipid peroxidation products: superoxide releases iron from iron-sulfur centered proteins, which then generates carbon-centered radicals that initiate lipid peroxidation, yielding breakdown products, such as 4-hydroxy-2-trans-nonenal, hydroxyhexenal malondialdehyde, most or all of which can activate the proton conductance of UCPs [75].

As far as the physiological role of the PUCP, the widespread presence of these proteins in climacteric or non-climacteric, as well as in thermogenic or non-thermogenic plants suggests that they may have functions other than heat production. In particular, ROS are one of the major components of a wide array of biotic and abiotic stresses, and mitochondria are a major intracellular source of ROS [79–81]; therefore, the ROS-induced activation of DWM-PUCP may play a role in plant defence against adverse environmental stimuli (see Sections 2.3 and 4).

### 2.3. Control of ΔΨ and ROS Production

**Figure 2.**
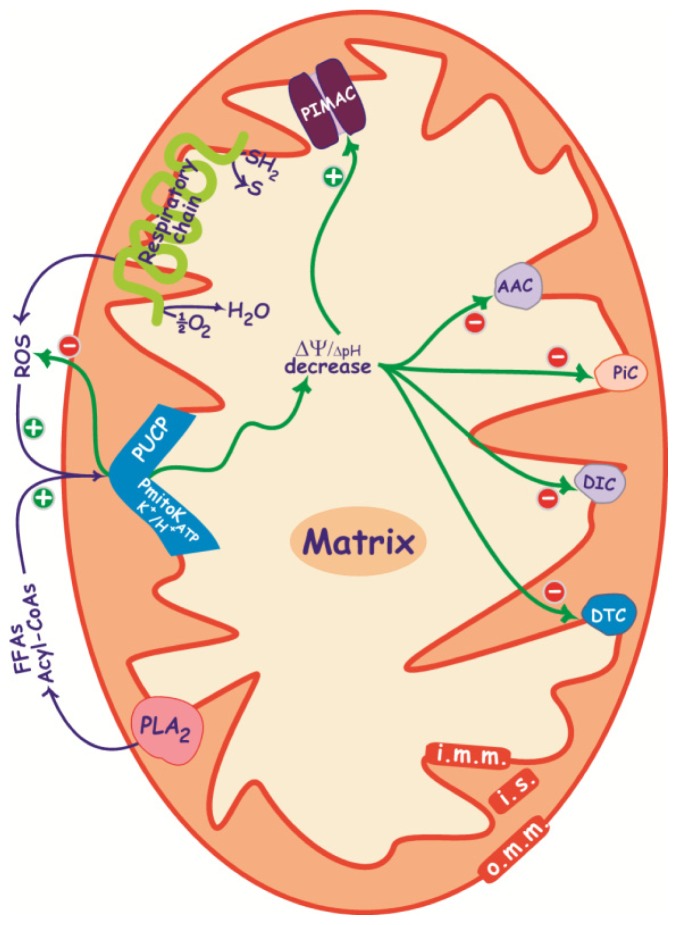
Effect of PmitoK_ATP_ and PUCP activity in DWM on ΔΨ/ΔpH, ROS production and other transport systems under hyperosmotic stress. Under stress an increase of FFAs due to PLA_2_ activity and probably of their acyl-CoA derivatives is observed, as well as an increased ROS production by respiratory chain and ATP synthesis inhibition, thus leading to PmitoK_ATP_ and PUCP activation. Once activated, both PmitoK_ATP_ and PUCP may cause ΔΨ and ΔpH decrease, which allows the prevention of further large-scale ROS production, according to a feedback mechanism. On the other side, ΔΨ and ΔpH decrease may modulate transport by impairing carriers and activating PIMAC. AAC, ADP/ATP carrier; DIC, dicarboxylate carrier; DTC, dicarboxylate-tricarboxylate carrier; PiC, Pi carrier; PIMAC, plant inner membrane anion channel; SH_2_, reduced substrates; S, oxidized substrates; PLA_2_, phospholipase A_2_; i.m.m., inner mitochondrial membrane; i.s., intermembrane space; o.m.m., outer mitochondrial membrane. ΔpH is represented in a smaller font size since it represents a lower component of pmf with respect to ΔΨ.

Skulachev’s theory postulates an important function for ROS-activated dissipative pathways, that is, to cause mild uncoupling in response to any ROS over-production, thus leading to a decrease in ΔΨ and a consequent reduction in further mitochondrial ROS generation according to a feedback mechanism [82,83]. In DWM, ROS-activated PmitoK_ATP_ and PUCP may cause a deep reduction of both ΔΨ and ΔpH (Figure 1), so exerting a positive strong control of dangerous mitochondrial ROS generation. In particular, it is well known that cellular ROS production increases as a result of plant exposure to various environmental stresses, thus inducing oxidative stress [79–81], and that mitochondria, in particular, increase their ROS generation under drought and salt stress [84]. Consistent with this, we observed an increase in the rate of superoxide anion production in mitochondria purified from osmotic- and salt-stressed durum wheat seedlings by about 40% under moderate stress conditions (germination in either 0.25 M mannitol or 0.125 M NaCl, respectively) and 120% under severe stress conditions (germination in either 0.42 M mannitol or 0.21 M NaCl, respectively) without significant differences between osmotic and salt stress [71]. Under the same conditions, we also observed an increase of FFAs and possibly of Acyl-CoA esters [61], these latter two deriving from the activation (up to about two times) of a mitochondrial phospholipase A_2_ (PLA_2_) [85], and a concurrent decrease of ATP [86,87], able to reduce the channel brake [87]. This change in the balance between modulators under stress favors the activation of the PmitoK_ATP_ (Figure 2); indeed, channel activity significantly increases under all stress conditions, and the activation is more evident under osmotic stress, with a more than 4-fold increase under severe conditions and a considerable increase even under moderate stress [71]. Once activated, the PmitoK_ATP_ may lower ΔΨ and ΔpH up to a complete dissipation, thus damping further large scale ROS production according to a feedback mechanism [88] (see also Figure 2). For example, the opening/closure of the PmitoK_ATP_ in a 100 mM KCl medium may cause a variation in the superoxide anion production up to about 35-fold [55]. This result was observed also in other plant mitochondria; indeed, in pea stem, the succinate-dependent H_2_O_2_ formation was progressively inhibited when mitochondria were resuspended in media containing increasing concentrations (from 50 to 150 mM) of KCl [89].

DWM-PUCP may cooperate with PmitoK_ATP_ to control ROS under stress condition. PUCP activation has been shown under osmotic and, in particular, under salt stress; the activation is stress-intensity dependent, and it is evident even under moderate stress [71]. We have also reported the capability of DWM-PUCP to reduce mitochondrial ROS generation in stressed DWM; in particular, a 70% reduction of the mitochondrial superoxide anion generation has been observed in the presence of externally added linoleate [71]. Interestingly, the increased PUCP functioning is not due to an increased *de novo* synthesis of the protein, as the expression levels of two PUCP-related genes are not affected by the stress imposition [68], but rather to an activation of the PUCP, able to quickly switch on the decrease on ΔΨ and ΔpH and, consequently, of ROS generation. Indeed, as already observed for PmitoK_ATP_, under stress conditions PUCP is strongly activated by endogenous ROS [71]; moreover, under hyperosmotic stress conditions, the above reported increase in the endogenous FFAs is able to increase PUCP functioning according to a second activation pathway [61].

Moreover, in contrast with unstressed DWM, DWM from hyperosmotically-stressed seedlings present a PUCP insensitive to ATP. A lower UCP sensitivity to nucleotides as a result of stress has been already observed in mitochondria from cold stressed rat skeletal muscle [90] and dehydrated slices of Jerusalem artichoke tubers [78]. In phosphorylating potato tuber mitochondria, the redox state of ubiquinone (Q), which can antagonistically be varied with antimycin A and *n*-butyl malonate, modulates inhibition of the palmitic acid-activated UCP-sustained H^+^ leak by GTP. This regulatory parameter enlightens on the most probable physiological role of UCP homologues, namely the control of energy metabolism balance of the cell. The UCP-sustained H^+^ leak can only be inhibited when Q reduction level is low, corresponding to a low availability in oxidizable substrates and a high ATP demand, in order to preserve ATP synthesis efficiency. Contrarily, the highly reduced state of Q, occurring at high substrate supply or low ATP demand, disables purine nucleotide inhibition, leading to an activation of UCP that consequently decreases the production of harmful ROS [91]. Interestingly, all trans-retinal-induced proton leak and 4-hydroxy-2-nonenal-induced proton leak in potato tuber mitochondria are also regulated by changes in the membranous Q reduction level. So, the inhibition of plant UCPs by purine nucleotides is under the control of mitochondrial Q reduction level, regardless of the type of activator involved (FFA or aldehyde).

These results are also in line with the frequently reported inefficiency of purine nucleotides in inhibiting the induced activity of UCPs [92]. In this regard, it is widely accepted that plants suffering from biotic or abiotic stress often show an over-reduction of electron carriers such as ubiquinone (Q), that causes electron leakage from the system with consequent ROS generation [93]; under these conditions, a decrease of PUCP sensitivity to purine nucleotides may help to better control ROS.

In the whole, modulation of PUCP is similar to the one of PmitoK_ATP_, thus suggesting a possible reinforcement of the feedback control described for potassium channel (Figure 2). So, DWM appear to be ready to dissipate pmf in order to avoid ROS over-production. Anyway, the loss of ΔΨ and ΔpH may modulate transports by impairing carriers, but at the same time also unlock PIMAC activity (Figure 2 and Section 3.3).

## 3. PIMAC, Carriers and Anion Transport in Energized and De-Energized DWM

### 3.1. PIMAC

The activity of inner membrane anion channels (IMAC) has been studied in liver and heart mitochondria [94]. IMAC catalyzes the electrophoretic uniport of a wide variety of physiological and nonphysiological, singly and multicharged, anions including chloride, sulfate, ferricyanide, bicarbonate, Pi, succinate, malate, citrate, ATP *etc.*, and it has been demonstrated to play crucial functions, such as the regulation of the mitochondrial volume and respiratory rates [95], and the control of the superoxide traffic among mitochondria [96].

In plant cells, the occurrence of the plant inner membrane anion channel (PIMAC) has been demonstrated in mitochondria from tubers of potato [40] and topinambur [39], and seedlings of durum wheat [39] and maize [38]. As previously observed for IMAC of mammalian mitochondria [97], evidence has been reported in seedlings of maize that a chloride channel protein, namely the ZmCLCc, can perform the PIMAC activity [42].

Similarly to mitochondria from tubers of potato and topinambur, DWM present a clearly evident swelling when suspended in iso-osmotic solutions of KCl or K^+^ salts of dicarboxylates, tricarboxylates, oxodicarboxylates and Pi (but not ATP), thus indicating that DWM are specifically permeable to both K^+^ and counteranions. As stated above, the K^+^ uniport in DWM is catalyzed by the PmitoK_ATP_ [44]; as far as the anion transport, it is strongly inhibited by propranolol, a specific IMAC and PIMAC inhibitor [40,98], thus showing that DWM–PIMAC can mediate the electrophoretic transport of these metabolically relevant anions. Chloride, succinate, malate, oxaloacetate, 2-oxoglutarate, citrate and *cis*-aconitate are transported at high rate by DWM-PIMAC, while Pi is also transported via PIMAC but at a rate 3- to 3.5-fold lower with respect to the other anions [39].

Regarding modulator sensitivity, DWM-PIMAC presents many of the characteristics of other animal and plant anion channels; in addition to the inhibition by propranolol, DWM-PIMAC is inhibited also by tributyltin. On the other hand, DWM-PIMAC, similarly to other plant anion channels, differs from IMAC in other important properties; notably, it is insensitive to mercurials and *N,N*′-dicyclohexylcarbodiimide at concentrations which inhibit IMAC [95]. As far as physiological modulators, DWM-PIMAC, similarly to PIMAC from potato and topinambur tubers, is not affected by Mg^2+^, which represents the most important inhibitor of IMAC [99]; on the contrary, it is inhibited, but not completely inactivated, by physiological concentrations of ATP [39,87], that acts at the outer side of the inner mitochondrial membrane; moreover, PIMAC is activated by alkaline pH, mainly at the matrix side [39]. To date, no information is available regarding the ATP sensitivity of the anion channels in mammalian mitochondria (it is known that ATP is a substrate of IMAC), while, interestingly, in yeast mitochondria matrix ATP was found to regulate, with a different mechanism, two anion channels with different conductance [100,101]. As for FFAs, several unsaturated and, to a lesser extent, saturated FFAs inhibit DWM-PIMAC, while acyl-CoAs, that were found to exert an inhibitory action on IMAC of rat liver mitochondria [102], had no effect on DWM-PIMAC; moreover, no effect on DWM-PIMAC, as well as on PIMAC of topinambur tubers, has been observed by ROS [39].

As expected, the PIMAC-mediated electrophoretic anion flux inside DWM is inhibited by high ΔΨ values, while it is fully promoted by a complete ΔΨ depolarization; nevertheless, at a ΔΨ of about 110 mV, an anion uptake through PIMAC, although inhibited, has been observed. In this regard, *in vivo* determination under different physiological conditions has shown the existence of low ΔΨ values in mitochondria from both plant [103] and animal [104–106] sources, thus suggesting that *in vivo* a PIMAC activity occurs at ΔΨ values that resemble the physiological ones, and that also little ΔΨ oscillations may greatly affect PIMAC activity.

**Figure 3.**
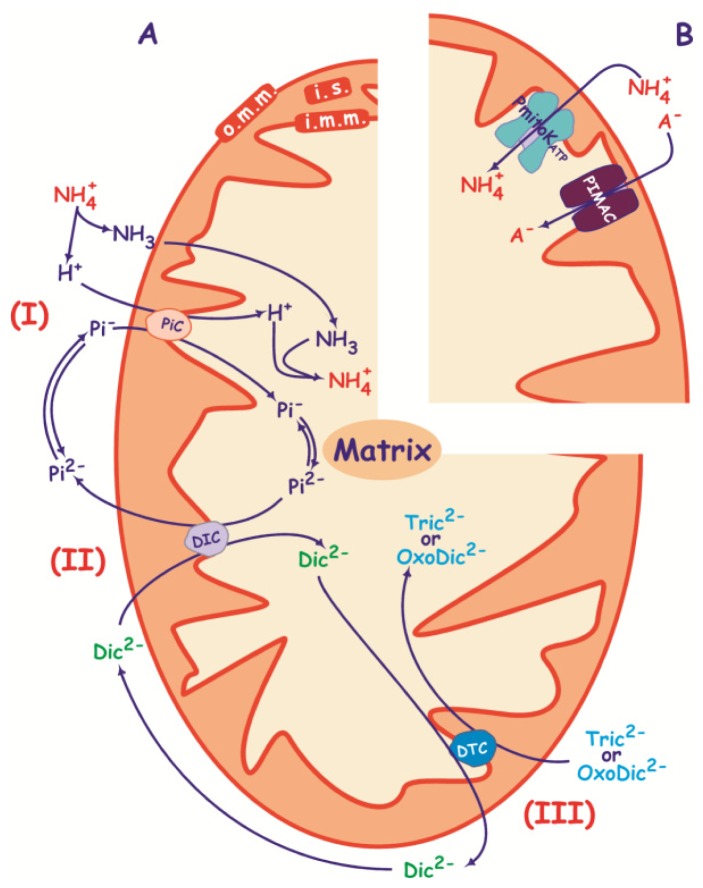
Mechanism of swelling in iso-osmotic solutions of NH_4_^+^ salts according to classical (**A**) and novel (**B**) modes. (**A**) In the first mode, NH_3_ diffuses across the membrane, down a concentration gradient (I). Inside the mitochondrion, NH_3_ associates with a proton to form NH_4_^+^. Pi^−^ is transported in symport with H^+^ on the Pi carrier (PiC); in the matrix Pi^−^ is in equilibrium with Pi^2−^ that exchanges for dicarboxylate (Dic^2−^) on the dicarboxylate carrier (DIC) (II), which in turn may exchange for oxodicarboxylate (OxoDic^2−^) or tricaboxylate (Tric^2−^) on the dicarboxylate-tricarboxylate carrier (DTC) (III); (**B**) In the second mode, anion (A^−^) uptake may be mediated by PIMAC, whereas NH_4_^+^ is transported via PmitoK_ATP_. i.m.m., inner mitochondrial membrane; i.s., intermembrane space; o.m.m., outer mitochondrial membrane.

Interestingly, DWM also swell in iso-osmotic solutions of NH_4_^+^ salts of chloride, malate, oxaloacetate, 2-oxoglutarate, citrate, *cis*-aconitate and Pi in a manner involving PmitoK_ATP_ and PIMAC. This is suggested by some observations. Firstly, none of the dicarboxylate, tricarboxylate and oxodicarboxylate swellings were found to be enhanced by Pi (or Pi plus malate), which is essential to trigger the swelling in intact mammalian/plant mitochondria according to the classical scheme reported in Figure 3A(I–III). NH_3_ diffusion across the membrane, Pi symport with proton via Pi carrier (I), dicarboxylate entry in exchange with Pi via the DIC (I and II) and tricarboxylate or oxodicarboxylate entry in exchange with the dicarboxylate via DTC in plants (I, II and III). Secondly, all these swellings are strongly inhibited by the PIMAC inhibitor propranolol, but not by the carrier inhibitor mersalyl. Finally, an increase in the rate of swelling in NH_4_^+^ salt is generally observed in the presence of the uncoupler carbonyl cyanide 4-(trifluoromethoxy)phenylhydrazone (FCCP) [39]; FCCP allows H^+^ entry into the matrix in competition with PiC; this activation is inconsistent with mechanisms in Figure 3A(I–III).

These findings allow explanation of some previously incomprehensible behaviors of plant mitochondria swelling observed in the past ([1], and references therein, [107]), thus also assessing that the classical interpretation of swelling experiments should be revised in plant mitochondria possessing active PmitoK_ATP_ and PIMAC. Swelling experiments of DWM in NH_4_^+^ salts show that transport mode of Figure 3B is much more active than that involving DIC and DTC [39]; in any case, Pi transport merits specification. It is not activated by FCCP, but even slightly inhibited; moreover, DWM incubation in the presence of NEM, a known inhibitor of Pi carrier ([108] and references therein) allows some (20%) inhibition. These observations suggest that both PIMAC and Pi carrier contribute significantly to the overall rate of swelling in NH_4_Pi in DWM and presumably in other plant mitochondria.

### 3.2. DWM Carriers

#### 3.2.1. ADP/ATP Carrier

A central role of mitochondria is to supply the cell with the ATP produced by oxidative phosphorylation. To do this, mitochondria have an ADP/ATP carrier (AAC), which exchanges cytosolic ADP with matrix ATP [109]; due to its central role in cell energetics, the AAC represents the most abundant carrier in the inner mitochondrial membrane [110]. The exchange involves ingoing ADP^3−^ and outgoing ATP^4−^, thus exploiting mitochondrial ΔΨ as driving force.

In plants, the existence of genes encoding AAC putative proteins has been reported for different species; in particular, three genes (*AAC1*, *AAC2* and *AAC3*) encoding different ACC isoforms and up to seven AAC-like genes have been identified in *Arabidopsis thaliana*, while two ACC genes and two AAC-like genes have been identified in the monocot species *Brachypodium distachyon* [7]. Investigations carried out on purified ACC from maize [111] and topinambur [112], as well as on recombinant AAC proteins and intact mitochondria from both potato and *Arabidopsis thaliana* [6] have revealed that, in analogy with the animal counterpart, the activity of the plant mitochondrial AAC is strongly reduced in the presence of bongkrekic acid and/or carboxyatractyloside (CATR), two specific AAC inhibitors [6]; moreover, compared to the nucleotide affinity of mammalian AACs (Km ranging between 40 and 140 μM), a significantly higher external affinity for ADP and ATP has been measured both in purified (Km ranging between 17 and 26 μM) and in recombinant AACs (Km ranging between 10 and 22 μM), as well as for adenine nucleotide transport in intact mitochondria (Km ranging between 1 and 2 μM).

As for DWM, evidence has been reported that these mitochondria are able to synthesise ATP from externally added ADP, and to export the newly synthesised ATP outside mitochondria [113]. In DWM translocation is the rate-limiting step of ATP appearance outside mitochondria; so, the ADP/ATP translocator can regulate the rate of ATP synthesis. Similarly to the adenine nucleotide transport from other plant mitochondrial systems, the ADP/ATP translocator in DWM displays a Km for the ADP significantly lower (21.7 ± 3.06 μM) than that reported for mammalian AACs. As far as sensitivity to inhibitors, the ADP/ATP exchange in DWM is inhibited by both atractyloside (ATR) and CATR, up to a complete stop at 3 μM CATR and 10 μM ATR [68]. In particular, incubation of DWM with 3 μM CATR causes a strong inhibition of the initial ATP efflux from mitochondria followed by a complete stop after few minutes, while incubation with 10 μM ATR completely prevents the ATP efflux. In addition to its main function of exchanging intra-mitochondrial ATP with the cytosolic ADP, evidence has been reported that the ADP/ATP carrier is also involved in the uncoupling effect of low concentrations of FFAs, raising the hypothesis that it is able to translocate the FFA anion by virtue of its role as a transporter of anionic substrates [114]. The effect is weak in liver and stronger in muscle mitochondria, which have a higher AAC content, but, in any case, it is weak as compared to uncouplers or to UCP [8]. Similar evidence has been reported also for mitochondria of different plant species. In pea stem and sunflower hypocotyls, CATR addition to mitochondria causes a weak (few mV) restoration of ΔΨ in organelles partially uncoupled by palmitate addition [115], but no ΔΨ recovery is observed at high FFA concentrations [116]. A more evident effect has been observed in durum wheat, in which the addition of CATR or ATR to mitochondria completely depolarised by a saturating linoleate concentration induces a ΔΨ recovery of about 25 mV [68]. So, although to a limited extent, the AAC carrier may cooperate with PUCP and PmitoK_ATP_ in the control of ΔΨ.

#### 3.2.2. Pi Carrier

Uptake of Pi into mitochondria through a Pi/OH^−^ antiport [117] or a Pi/H^+^ symport [118] is essential for the oxidative phosphorylation of ADP to ATP [119]. Inside the matrix, Pi plays also additional roles, since it allows the uptake of other metabolites via exchange mechanisms catalysed by other mitochondrial transporters [2,5]. Recently, involvement of mitochondrial Pi carrier (MPC/PiC) in the Pi activation of the permeability transition pore opening has been also postulated [120]. The electroneutral Pi transport is driven by the pH gradient across the membrane, which is generated by the mitochondrial electron transport chain, to accumulate high levels of Pi in the matrix.

In plants, the first gene encoding a putative PiC has been cloned from birch (*Betula pendula* Roth) [121]. Afterwards, cDNAs encoding the mitochondrial PiC have been isolated from various species including *Arabidopsis thaliana*, in which three PiC genes have been identified, *AtMPT1*, *AtMPT2* and *AtMPT3*, also named *AT2*, *AT3*, *AT5* and *PHT3;3*, *PHT3;2*, *PHT3;1* ([122], and references therein). The existence of PiC activity in plant mitochondria has been demonstrated by means of biochemical approaches; indeed, evidence has been reported that soybean recombinant PiC protein reconstituted in liposomes presents high Pi transport activity inhibited by NEM, like those of mammalian Pi transporters [123]. As far as *Arabidopsis thaliana*, the functional complementation strategy has demonstrated that two putative PiC proteins can restore Pi transport activity in a yeast mutant lacking the endogenous PiC [28].

As far as DWM, these mitochondria possess PiC since they swell in an iso-osmotic solution of NH_4_Pi in a NEM-sensitive way [39,44]. Consistently, Pi addition to succinate-energized mitochondria causes a slight increase of ΔΨ [44], as expected if Pi enters via proton-compensated symport [124,125].

#### 3.2.3. Succinate/Malate Antiport

Evidence has been reported in the literature supporting the idea that, among the members of the MCF, there are two types of carrier proteins potentially involved in succinate transport in plant mitochondria: the SFC and the DICs.

In the *Arabidopsis thaliana* genome, a gene encoding a SFC, named SFC1 has been identified by the functional complementation strategy in the yeast mutant, characterized by a deletion of the gene *acr1* that encodes this transporter [16]. ACR1 has the highest affinities for succinate and fumarate, but also recognizes a broad spectrum of di- and tricarboxylates, such as oxoglutarate, oxalacetate, malate, phosphoenolpyruvate, citrate or isocitrate [15]. The substrate specificity of the recombinant plant SFC1 has not been analyzed in detail to date, but it has been suggested that the plant carrier SFC1 may act in a similar way to the yeast ACR1.

As far as DICs, three potential homologues of mammalian and yeast mitochondrial DICs, designated as DIC1, DIC2 and DIC3, have been identified in *Arabidopsis thaliana* [13]. Characterization of the recombinant and reconstituted DIC isoforms has revealed that they present characteristics that resemble those of the non-plant organisms. Notably, similarly to animal and yeast, plant DICs transport a wide range of dicarboxylic acids including succinate and malate, and their activity is affected by phenylsuccinate, an impermeable dicarboxylate analogue, which has been found to markedly inhibit the activity of DIC1 and DIC2 and, to a lesser extent, that of DIC3 [13]. It is likely that DICs have an anaplerotic function, that allows transport of dicarboxylic acids into mitochondria, which are then used as respiratory substrates; this function is supported by evidence that succinate and malate oxidation by plant mitochondria is inhibited by DIC inhibitors ([13], and references therein).

Consistent with these findings, we have reported evidence that succinate is oxidized by DWM and that in these mitochondria transport across the inner mitochondrial membrane represents the limiting step of succinate oxidation; also in this case, competitive inhibition of the transport by phenylsuccinate (Ki ~0.63 mM, [126]) suggests an involvement of DICs. A similar result has been obtained in mitochondria from potato cell culture, in which succinate has been found to enter mitochondria in exchange with malate [53], thus further supporting the idea that, in plant mitochondria, succinate transport may occur via DICs.

Our findings show that in DWM succinate uptake is inhibited by ROS after a few minutes of exposure; indeed, in the presence of the superoxide anion producing system, xanthine/xanthine oxidase system, a mixed inhibition has been observed in the succinate-induced ΔΨ generation [126]. The inhibition of succinate transport in DWM is not dependent on the interaction between ROS and thiol groups; on the basis of the mixed nature of the inhibition, it is feasible that the inhibition could depend on the interaction of ROS with a variety of mitochondrial components, including protein carrier domains and membrane lipids close to the carrier. As far as the physiological implications of such an inhibition, it is likely that the impairment of succinate translocator by ROS may act as a defence mechanism by reducing the energy metabolism and the consequent large-scale ROS generation by mitochondria during environmental/oxidative stress in plants.

Interestingly, in DWM, succinate/malate exchange consists of two phases: the first phase mediated exclusively by PIMAC, that, at low ΔΨ, favours the accumulation of succinate inside mitochondria with consequent oxidation to malate as well as ΔΨ generation; this triggers the second phase in which succinate is exchanged with malate through the DIC; during this second phase the contribution of PIMAC should be negligible due to the high ΔΨ. So, PIMAC and energy-dependent carriers may cooperate with each other. This result is not unique; the existence of a first phase of substrate uptake followed by a second phase of exchange has been already proposed as a general mechanism that operates in several transport systems both in animal [127] and plant mitochondria [43].

#### 3.2.4. Malate/Oxaloacetate Antiport

In addition to their function in the exchange of organic acid intermediates that connects the Krebs cycle with diverse metabolic processes (*i.e.*, gluconeogenesis, glyoxylate cycle, synthesis of amino acids and nucleic acids), DICs may function as malate/oxaloacetate shuttle providing other cell compartments with reducing equivalents [13]. Because of the redox gradient between the NADH/NAD^+^ systems in the mitochondria and cytosol, the malate/oxaloacetate shuttle is thought to be more aligned energetically to export NADH from mitochondria than to import it. This could be important in photosynthetic tissues, in which the mitochondrial malate/oxaloacetate shuttle is involved, together with the chloroplastic malate valve, in the shift of malate to peroxisomes for the production of the NADH needed for the synthesis of glycerate from hydroxypyruvate in the photorespiratory cycle [128].

We have reported evidence that in DWM, as well as in potato cell mitochondria, a malate/oxaloacetate exchange occurs [129] and that this exchange is inhibited in a competitive manner by phenylsuccinate and butylmalonate, thus suggesting that it is due to a malate/oxaloacetate antiporter rather than to the combined activities of possible electrogenic uniporters of malate and oxaloacetate linked to each other for the charge compensation as reported by Zoglowek *et al.* [107]. Interestingly, our findings demonstrate that, both in durum wheat and potato cells, the malate/oxaloacetate antiporter, together with the cytosolic and mitochondrial malate dehydrogenase (cMDH and mMDH, respectively), allows the operation of a malate/oxaloacetate shuttle, which is responsible for the transfer of reducing equivalents inside mitochondria: malate enters mitochondria in exchange with endogenous oxaloacetate; once inside the matrix, malate is oxidized via the very active mMDH to oxaloacetate [113,130], which, in turn, can exit mitochondria, where it is reduced by the cMDH, with NADH consumption. Once inside mitochondria, the NADH can be oxidized by the respiratory chain-linked NADH dehydrogenase. Our results indicate that the antiporter regulates the rate of the overall operation of the malate/oxaloacetate shuttle.

At the physiological level, the malate/oxaloacetate shuttle plays a major role in the oxidation of cytosolic NADH both in durum wheat and potato cells. Indeed, on the basis of the kinetic parameters of the antiporter, of the external NADH dehydrogenase (NADH DH_ext_) and of the cMDH, and assuming that the cytosolic NADH concentration is about 1 μM [131], we conclude that the shuttle-dependent oxidation rate is about 100- and 10-fold higher than that due to the NADH DH_ext_ in durum wheat and potato cells, respectively. Both durum wheat and potato cells present a very high cMDH reaction rate, which does not limit the maximal rates of malate/oxaloacetate exchange even in the presence of physiological concentration of the product pair (1 mM malate and 0.5 mM NAD^+^). It is feasible that the contribution of the NADH DH_ext_ to NADH oxidation could increase under conditions in which a decrease in the cytosolic reduction potential occurs; for example, as observed under environmental stress, when plants suffer over-reduction of the carriers of the photosynthetic electron transport [132,133].

Due to the specificity of the cytosolic MDH for NADH, the malate/oxaloacetate shuttle cannot oxidize NADPH, whose oxidation occurs via the external NADPH dehydrogenase(s).

#### 3.2.5. Proline Transport

Proline is an amino acid that serves different functions in the plant cell. Besides its structural role as component of proteins, proline also acts as a compatible solute and its accumulation to high levels in response to the imposition of a wide range of biotic and abiotic stresses represents one of the major strategies to support the plant cell functionality under adverse conditions [134]. In addition, proline plays other roles during stress, *i.e.*, as antioxidant, metal chelator and signaling molecule. Evidence exists that the cellular levels of proline are regulated by the rate of both biosynthesis and degradation ([135], and references therein). Moreover, due to the compartmentation of the anabolic and catabolic processes in chloroplast and mitochondria, respectively, regulation of the intracellular proline transports cannot also be excluded; unfortunately, to date, little information is available in plants about the identity and the regulation of transporters responsible for the flux of proline into and out of specific intracellular compartments.

Mitochondria are devoted to proline metabolization to glutamate via proline dehydrogenase and Δ^1^ pyrroline-5-carboxylate dehydrogenase, which implies a proline flux from the cytosol to the matrix. In this regard, evidence has been reported, in DWM, about the existence of two different carriers for proline: a proline uniporter, which facilitates proline transport into the mitochondrial matrix; and a proline/glutamate antiporter, which appears to have an important role in the proline/glutamate exchange between the mitochondrial matrix and the cytosol [43]. However, the genes encoding proline transporters have not been identified in any plant [2].

Evidence that the two carriers differ from each other derives from their kinetic properties. In particular, the two transporters show a different affinity for proline, with a Km value of 2 and 8 mM for the uniporter and the antiporter, respectively. Moreover, the uniporter is inhibited by the non-penetrant thiol reagent mersalyl, but it is insensitive to the penetrant thiol reagent NEM, which, in contrast inhibits the antiporter; this suggests that the uniporter contains thiol/s in a hydrophilic environment whereas thiol/s belonging to the antiporter resides in the hydrophobic protein domain. The occurrence of two separate carriers for proline in DWM is further confirmed by the time course of ΔΨ generation, which starts immediately after the addition of proline to DWM, and that of the glutamate efflux, which starts about 1 min after the addition of proline, that is after reaching the maximum ΔΨ. On the basis of these findings the following mechanism can be proposed: the net uptake of proline occurs, and then, as a result of proline catabolism inside DWM [136], glutamate is generated and is then exported outside mitochondria in exchange with further proline via the proline/glutamate antiporter. Notice that these findings are consistent with the occurrence of the proline/glutamate shuttle of cytosolic NADH. This proposed shuttle might be considered to join the malate/oxaloacetate shuttle [129] in transferring the reducing equivalents from cytosol to mitochondria and *vice-versa*.

In addition to protein-mediated transports, an additional diffusion process has been observed in spinach leaf mitochondria [137]; this latter could contribute to proline uptake inside mitochondria under stress conditions, when high proline concentrations in the cytosol may be reached.

### 3.3. Anion Transport in De-Energized DWM

**Figure 4.**
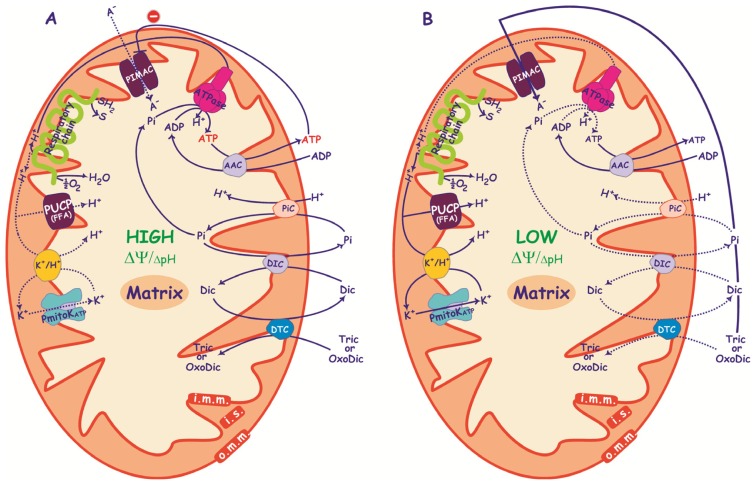
DWM uptake of Pi, dicarboxylates (Dic), oxodicarboxylates (OxoDic) and tricarboxylates (Tric) under conditions of high (**A**) and low (**B**) ΔΨ/ΔpH and ATP. (**A**) Under conditions in which high ΔΨ/Δ pH are generated by the respiratory chain and ATP is synthesized at a high rate, PIMAC is inactive due to ATP inhibition and ΔΨ regulation; metabolically relevant anions are transported by the specific energy-dependent carriers (AAC, PiC, DIC and DTC); (**B**) When ΔΨ/ΔpH are lowered by PmitoK_ATP_ and PUCP functioning and ATP is synthesized at lower rate, anion transport can be mediated by PIMAC. PiC, Pi carrier; DIC, dicarboxylate carrier; DTC, dicarboxylate-tricarboxylate carrier; AAC, ADP/ATP carrier; SH_2_, reduced substrates; S, oxidized substrates; i.m.m., inner mitochondrial membrane; i.s., intermembrane space; o.m.m., outer mitochondrial membrane. ΔpH is reported in a smaller font since it represents a lower component of pmf with respect to ΔΨ. Dotted lines represent low active pathways.

In energized mitochondria, PIMAC functioning should be inhibited by ATP and high ΔΨ values; under these conditions ΔΨ allows for functioning of the AAC and ΔpH represents the driving force for the PiC, that triggers DIC and DTC activity (Figure 4A). The set-up is different in de-energized mitochondria. PIMAC should play a role that gradually increases under conditions in which mitochondria are progressively de-energized by replacing or integrating the operation of anion carriers to ensure the exchange of metabolites between mitochondria and other organelles. This is likely to occur in DWM in the light of the presence of powerful energy-dissipating systems such as PmitoK_ATP_ and PUCP (see above) as well as alternative oxidase (AOX) [130], whose functioning under abiotic stress conditions allows the control of mitochondrial ROS generation.

As already shown in Figure 2, under stress conditions the decrease in both ΔΨ and ATP activates PIMAC, while the increase in FFAs may put a brake on excess activation, which might cause swelling and rupture of outer mitochondrial membrane. At the same time, the decreased ΔΨ and ΔpH, as well as the increased ROS generation, may impair anion carriers; as a consequence, the anion transport across the inner mitochondrial membrane may shift from the energy-dependent anion carriers toward the electrophoretic flux through the PIMAC (Figure 4B).

Consistently, evidence for a role of PIMAC in plant response to abiotic stress has been also reported in maize seedlings; the analysis of three maize populations differing in terms of cold tolerance highlighted a relationship between the PIMAC activity kinetics and the level of cold tolerance [38]; in addition, in the same plant material, the level of the ZmCLCc protein, responsible for the PIMAC activity in maize seedling, was found to be higher in cold stressed than in non-stressed growing conditions [42].

The picture reported in Figure 4B raises two main questions: how ATP may be synthesized at low driving force and how it may be exported outside mitochondria, as PIMAC cannot transport ATP (see Section 3.1).

**Figure 5.**
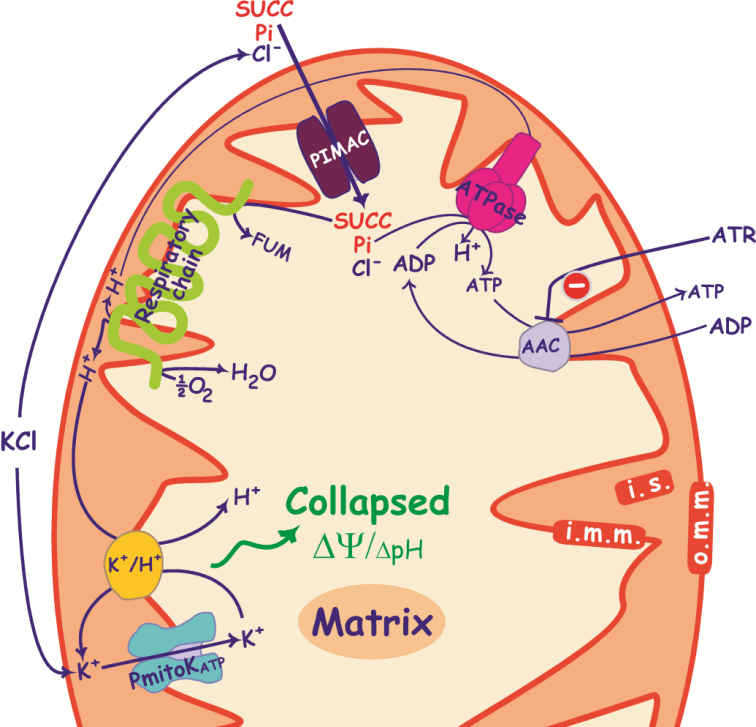
Uptake of Pi and succinate (SUCC), and ADP/ATP exchange in DWM fully de-energized by PmitoK_ATP_. SUCC-oxidising DWM added with KCl at high concentration present a strongly reduced ΔΨ/ΔpH due to PmitoK_ATP_ functioning. Under a number of experiments, a complete ΔΨ/ΔpH collapse was even observed. Unexpectedly, in the absence of a measurable pmf, DWM regularly synthesize ATP; under this condition, SUCC and Pi uptake may occur via PIMAC, whereas the ADP/ATP exchange occurs via AAC despite the absence of ΔΨ (for details see the text). AAC, ADP/ATP carrier; FUM, fumarate; ATR, atractyloside; i.m.m., inner mitochondrial membrane; i.s., intermembrane space; o.m.m., outer mitochondrial membrane. ΔpH is reported in a smaller size since it represents a lower component of pmf with respect to ΔΨ.

In this regard, we have reported evidence in a recent paper that ATP synthesis and efflux may be maintained when de-energization of succinate oxidising DWM is obtained via PmitoK_ATP_ in a high KCl medium [55]. In particular, under these conditions DWM are unexpectedly fully coupled as they preserve ADP/O ratio and do not change their rate of ATP synthesis and efflux despite complete pmf decrease. A possible explanation for this apparently inconsistent behaviour resides in the inhibition of the PmitoK_ATP_ by ATP; in practice, the channel may strongly lower measurable bulk phase pmf, but ATP inhibition represents a brake, so it appears unable to fully compete with ATP synthase for protons, thus preserving a latent proton movement non-classically detectable [55,138]. As for transports, it is likely that succinate and Pi transport may mainly occur via PIMAC (Figure 5). On the other hand, ADP/ATP exchange resulted in full inhibition by ATR [55] (see also Figure 5); this surprising finding strongly suggests that adenine nucleotide movement is dependent on the ADP/ATP carrier despite the absence of ΔΨ. To date, how this transport may actively occur in fully de-energized DWM remains to be established. Anyway, these *in vitro* results show that mitochondria are able to maintain transport activity even in the absence of any measurable driving force.

It should be also underlined that in potato tuber mitochondria, under phosphorylating conditions, the coupling parameters, *i.e.*, the ADP/O ratio and the respiratory control ratio, were unchanged in mitochondria respiring either in the absence or in the presence of 100 mM KCl (at constant osmolarity). These measurements provide direct evidence against significant uncoupling as a consequence of K^+^ channel opening in mitochondria of potato tubers [41].

## 4. Conclusions

Studies about DWM have shed some light about the interrelationship between transport systems and pmf in plant mitochondria. In particular, PmitoK_ATP_ and PUCP may actively dissipate pmf, but, unexpectedly, de-energization of mitochondria *in vitro* does not impair metabolite transport. Indeed, PIMAC may cooperate with carriers, and the ADP/ATP carrier may show activity also in the absence of measurable ΔΨ. These findings may have a relevant physiological importance. In this regard, evidence has been reported that mitochondria depolarization is not unusual *in vivo*. As described above, dissipative systems may lower ΔΨ to avoid excess ROS production under environmental stresses. Moreover, determination of the *in vivo* dynamics of individual mitochondrial membrane potentials in *Arabidopsis thaliana* roots has demonstrated that plant mitochondria undergo sporadic and rapid cycles of partial dissipation and restoration of ΔΨ [139]. Probably, PmitoK_ATP_ may also induce partial ΔΨ dissipation *in vivo*, as a result of a balance between positive and negative modulators. Under these ΔΨ dissipation cycles cooperation between PIMAC and carriers might ensure mitochondrial metabolism *in vivo*. Similarly, animal mitochondria are reported to be exposed to complete depolarization as a consequence of spontaneous opening and closure of the permeability transition pore [140]. Finally, it should be considered that ΔΨ values measured *in vivo* are generally low. In animals, Zhang *et al.* [106] measured a mitochondrial ΔΨ of about 105 mV in fibroblasts and 81 mV in neuroblastoma cells; in perfused hearts [104] and single hepatocytes [105] values of 100–140 mV were measured under different metabolic conditions. This is true also in plant mitochondria, as Igamberdiev and Kleczkowski [103] estimated a mitochondrial ΔΨ of 70–95 mV in barley leaf protoplasts under different physiological conditions. So, the transport systems in plant mitochondria must be active under low driving force also *in vivo*. Further studies are required to fully understand this behaviour.
